# Decoding Bleeding Risks and Survival in Patients Undergoing Percutaneous Coronary Intervention on Antiplatelet Therapy

**DOI:** 10.1016/j.jacasi.2025.05.017

**Published:** 2025-07-29

**Authors:** Chun-Chin Chang, Andrew Kei-Yan Ng, Norihiro Kogame, Po-Hsun Huang, Byeong-Keuk Kim, Robert-Jan M. van Geuns

**Affiliations:** a Division of Cardiology, Department of Medicine, Taipei Veterans General Hospital, Taipei, Taiwan; b Cardiovascular Research Center, National Yang Ming Chiao Tung University, Taipei, Taiwan; c Gleneagles Hospital Hong Kong, Wong Chuk Hang, Hong Kong; d Division of Cardiovascular Medicine, Toho University Ohashi Medical Center, Tokyo, Japan; e Division of Cardiology, Severance Cardiovascular Hospital, Yonsei University College of Medicine, Seoul, South Korea; f Department of Cardiology, Radboud University Medical Center, Nijmegen, the Netherlands

**Keywords:** coronary artery disease, dual antiplatelet drug, high bleeding risk, percutaneous coronary intervention

## Abstract

This comprehensive review focuses on bleeding risk and outcomes in patients with coronary artery disease undergoing percutaneous coronary intervention (PCI) with antiplatelet therapy. Bleeding prevalence varies widely, ranging from 1% to 10% in clinical trials and 2.8% to 11% in real-world studies, with a significant impact on mortality. The Academic Research Consortium for High Bleeding Risk criteria are well-validated, classifying approximately 50% of patients as high bleeding risk, who subsequently experience higher post-PCI bleeding rates. Key risk factors include advanced age, chronic kidney disease, and multiple comorbidities. This review also explores bleeding event definitions, risk stratification methods, and the clinical consequences of bleeding. The strong association between bleeding and mortality after PCI underscores the importance of vigilant monitoring and tailored management strategies.

## Highlights

• Major bleeding events in patients with CAD on antiplatelet therapy significantly increase mortality risk, highlighting the need for careful risk management.
• East Asian patients display higher bleeding and lower ischemic risks compared with Western population, necessitating tailored antithrombotic therapies.
• Patients with both high bleeding and ischemic risks require careful balancing of antiplatelet therapy to optimize outcomes, with studies showing clopidogrel monotherapy can reduce bleeding without compromising ischemic protection.

Dual antiplatelet therapy (DAPT) is an established standard of care for patients undergoing percutaneous coronary intervention (PCI) for either chronic coronary syndrome (CCS) or acute coronary syndrome (ACS). The American College of Cardiology/American Heart Association and European Society of Cardiology Guidelines for the management of ACS provide extensive recommendations for the treatment of ACS.^1^^,^^2^ However, the use of antiplatelet treatment does not come without a cost. Although antiplatelet therapy reduces thrombotic events, it increases the risk of bleeding.^3^ The risk of bleeding is amplified in patients with criterion of high-bleeding risks (HBRs) such as age ≥75 years, taking anticoagulants, with chronic kidney disease, and so on.

Most troubling is that the occurrence of major bleeding was linked to an approximately 3-fold increase in the risk of mortality. Thus, selecting an optimal antiplatelet therapy for individual patients to prevent thrombotic events while balancing the risk of bleeding is fundamental.^4^^,^^5^ Even minor bleeding can lead to the interruption of DAPT, which might have significant implications for the patient's outcomes.^6^

The approach to managing patients receiving DAPT has historically been centered on minimizing thrombotic risks, given the importance of preventing ischemic complications. However, there has been a shift in recent years toward a more balanced and comprehensive strategy that takes bleeding risk into priority consideration.^7^ In essence, the shift represents a more patient-centric perspective in the management of DAPT, acknowledging that a 1-size-fits-all approach would not be ideal in optimizing both ischemic and bleeding outcomes.

This review will discuss a wide array of evidence, including the prevalence of bleeding in patients receiving antiplatelet therapy after intervention, the most probable timing of major bleeding, the risk stratification based on clinical features, the proposed mechanism of action on consequence of bleeding, and most importantly, the impact of bleeding on the rate of mortality.

## Prevalence of Bleeding Events in Patients With CAD

In the 2019 consensus document from the Academic Research Consortium for High Bleeding Risk (ARC-HBR), the 1-year overall bleeding rates in trials of antiplatelet therapy were analyzed, and it was reported that the overall bleeding ranged from 0.3% to 2.8 %, with the incidence of bleeding in majority of the studies fell into 2%.^8^

A systematic review investigated the incidence and prognostic impact of postdischarge bleeding after ACS in the outpatient setting by including 53 studies with over 700,000 participants.^4^ The observational studies showed that major bleeding events occurred in approximately 1.29% to 3.25% of patients within the first 12 months after discharge. Incidence of minor bleeding and nuisance bleeding were even more common, occurring in approximately 6.56% to 10.6% and 21.9% to 37.5% of patients, respectively.^4^ The data indicates that the highest occurrence of bleeding was observed during the first 3 months following hospital discharge for ACS. A significant proportion of these postdischarge bleeding events were nuisance bleeding, primarily presenting as ecchymosis, and petechiae. However, recent evidence elucidated that nuisance bleeding is anything but inconsequential to the clinical outcomes

In general, clinical trials reported rates of major bleeding ranging from 1% to 10%.^9^, ^10^, ^11^ On the other hand, observational studies in real-world clinical settings indicated a higher incidence of major bleeding (2.8%-11.0%).^4^^,^^11^ The prevalence and incidence of bleeding varied globally, by population demographics and health care practices. These findings indicate the need in minimizing bleeding complications to improve overall care and outcomes of patients undergoing PCI.

## Bleeding Event Definition

Various bleeding definitions have been used in clinical trials and registries (Table 1). The use of different definitions for bleeding events in clinical research has posed challenges when the safety and efficacy of antiplatelet therapies are appraised based on the results of different trials. For instance, TIMI definition assesses bleeding severity by considering both laboratory parameters (eg, hemoglobin drop) and clinical events (eg, transfusion, intracranial bleeding).^12^ The Global Use of Strategies to Open Occluded Coronary Arteries (GUSTO) definition classifies bleeding as severe, moderate, or mild based on hemodynamic impact and the need for intervention.^13^ Platelet Inhibition and Patient Outcomes (PLATO) criteria classified bleeding into minor, major, and life-threatening categories.^14^ Different classification schemes employ distinct criteria for severity and types of bleeding, leading to inconsistencies in reporting and interpreting outcomes. These disparities hinder the comparability of the results of clinical trials and complicate the development of standardized treatment guidelines. Due to the lack of standardized bleeding definitions in clinical trials and the need of a consistent approach to collect bleeding data and adjudicate events, the Bleeding Academic Research Consortium (BARC) was developed to standardize bleeding definitions with hierarchical grading system.^15^^,^^16^ ARC-BARC divides bleeding into 5 main categories ranging from minor, nonactionable bleeding (type 1) to fatal bleeding (type 5), with specific subcategories detailing the severity and clinical impact. Minor bleeding (types 1 and 2) includes events that may require medical attention but do not lead to significant intervention or hospitalization. Major bleeding is defined as BARC type 3 or above. Type 3 encompasses more severe events, with subcategories based on hemoglobin drop (of 3-5 g/dL or at least 5 g/dL), need for transfusion or surgical intervention for control, and specific conditions like intracranial hemorrhage or intro-ocular bleed compromising vision. Type 4 includes bleeding related to coronary artery bypass graft (CABG) surgery, while type 5 distinguishes between probable fatal bleeding (5a) and definite fatal bleeding (5b).

**Table 1:** Comparison of Bleeding Categories

BARC^15^	TIMI^48^	GUSTO^49^	PLATO^50^	CURRENT OASIS^51^	REPLACE2^52^	STEEPLE^53^	ISTH^54^
1 Not actionable	Minimal	Mild	Minimal	Other minor	Minor	Minor	Minor
2 Overt, actionable; needing medical intervention/evaluation, prolonged hospitalization	Minor		Minor	Minor			
3a Overt; Hg drop 3-5 g/dL; transfusion		Moderate	Other major	Other major	Major	Major	Major
3b Overt; Hg drop *>*5 g/dL; needing surgery; vasoconstriction	Major	Severe	Major	Severe			
3c Intracranial; intraocular (vision)							
4 CABG related; reoperation; 5-U transfusion; chest tube placement							
5 Probable/confirmed fatality							

BARC = Bleeding Academic Research Consortium; CURRENT OASIS = Organization for the Assessment of Strategies for Ischemic Syndromes; GUSTO = Global Use of Strategies To Open Coronary Arteries; IISTH = International Society on Thrombosis and Haemostasis; PLATO = Platelet Inhibition and Patient Outcomes; REPLACE2 = Randomized Evaluation in PCI Linking Angiomax to Reduced Clinical Events; STEEPLE = Safety and Efficacy of Enoxaparin in Percutaneous Coronary Intervention Patients, an International Randomized Evaluation; TIMI = Thrombolysis In Myocardial Infarction.

## Bleeding Risk Stratification

As patients with high bleeding risk are more susceptible to adverse outcomes, including potentially death, identifying patients with high-bleeding risk is paramount in the management of patients receiving antiplatelet therapy following PCI.^8^ Recognizing these patients allows clinicians to tailor the DAPT regimen, such as a shorter duration of DAPT or the use of different antiplatelet agents to balance the risk of bleeding while maintaining efficacy in the prevention of thrombotic events. Therefore, tools for bleeding risk stratification such as bleeding risk scores may be used to guide the use of antiplatelet agents.

A variety of risk scores, such as the DAPT score, the PARIS score (Patterns of Non-adherence to Antiplatelet Regimen in Stented Patients), and the PRECISE-DAPT score (Predicting Bleeding Complications in Patients Undergoing Stent Implantation and Subsequent Dual Antiplatelet Therapy), have been developed to guide the use of antiplatelet therapies by assessing and stratifying patients’ risk of bleeding complications (Table 2).

**Table 2:** Different Scoring Systems for Predicting High Bleeding Risk

	CRUSADE^55^	ACUITY^56^	REACH^57^	DAPT^58^	PARIS^59^	PRECISE-DAPT^60^	BleeMACS^61^	CREDO-Kyoto^24^	CARDIAC^62^	ARC-HBR (8)	J-HBR ^33^	PRECISE-HBR^25^
Year	2009	2010	2010	2016	2016	2017	2018	2018	2022	2019	2020	2025
Predicted outcome	In-hospital major bleeding	30-day major bleeding	Major bleeding at 2 y	Major bleeding at 1 y	Major bleeding at 2 y	Out-of-hospital major bleeding	Major bleeding at 1 y	Moderate or severe bleeding at 3 y	Major bleeding at 1 y	Major bleeding at 1 y	Major bleeding at 1 y	Major bleeding at 1 y
No. of variables	8	7	9	9	6	5	7	7	5	20	20	7
Score range	1-100	−5 to 52	0-23	−2 to 10	0-14	0-100	0-80	0-11	0-16	Binary	Binary	0-70

**Variables for scoring assessment**												

Age		**✓**	**✓**	**✓**	**✓**	**✓**	**✓**		**✓**	a	a	**✓**
Low body weight, frailty											b	
CKD	**✓**	**✓**			**✓**	**✓**	**✓**	**✓**	**✓**	a^,^^b^	a^,^^b^	**✓**
Liver disease										b	b	
Blood test (Ht/WBC/PLT)	**✓** (Ht)	**✓** (WBC)				**✓** (WBC)		**✓** (PLT)	**✓**	b (PLT)	b (PLT)	**✓** (WBC)
Anemia		**✓**			**✓**	**✓**	**✓**		**✓**	a^,^^b^	b / a	**✓**
Heart failure	**✓**		**✓**					**✓**			b	
Vascular disease		**✓** (STEMI)	**✓** (PAD)	**✓** (MI)			**✓**	**✓** (PVD)			b (PVD)	
Prior vascular disease	**✓**			**✓** (PCI or MI)				**✓** (MI)		b (ICH, bVAM or IS) / a (IS)	b (ICH or IS) / a (IS)	
Prior bleeding						**✓**	**✓**			a^,^^b^	a^,^^b^	**✓**
Bleeding diathesis										b	b	
Anticoagulant			**✓**		**✓**				**✓**	b	b	**✓**
NSAID										a	a	
Active cancer							**✓**	**✓**		b	b	
Major/recent surgery										b	b	
BMI					**✓**							
Gender/sex	**✓**	**✓**										
Diabetes	**✓**		**✓**	**✓**								
Smoking			**✓**	**✓**	**✓**							
Heart rate	**✓**							**✓** (AF)				
Hypertension			**✓**				**✓**					
Blood pressure	**✓**											
Others		Types of antithrombotic medications	Hypercholesterolemia	• Paclitaxel-eluting stent • Stent diameter <3 mm • Vein graft stent								At least 1 of the remaining ARC-HBR elements (except previous stroke)

**Scoring Classification**												

	Very low: ≤20 Low: 21-30 Moderate: 31-40 High: 41-50 Very high: >50	Low risk: <10 Moderate risk: 10-14 High risk: 15-19 Very high risk: ≥20	Low risk: 0-6 Intermediate risk: 7-8 Intermediate-high risk: 9-10 High risk: 11-21	Long DAPT: score ≥2 Standard DAPT (12 mo): score <2	Low risk: 0-2 Intermediate risk: 4-7 High risk: ≥8	Not HBR: <25 HBR: ≥25	Very low risk: ≤7 Low risk: 8-16 Moderate risk: 17-24 High risk: ≥25	Low: 0 Intermediate: 1-2 High: 3-11	Cutoff for major bleeding prediction: ≥5	Not HBR: Not meeting HBR criteria HBR: ≥1 major or 2 minor criteria	Not HBR: Not meeting HBR criteria HBR: ≥1 major or 2 minor criteria	Not HBR: ≤22 HBR:23-26 Very high ≥27

**✓**Variable included for scoring assessment.

ACUITY = Acute Catheterization and Urgent Intervention Triage Strategy; ACS = acute coronary syndrome; AF = atrial fibrillation; bVAM = brain arteriovenous malformations; BleeMACS = Bleeding Complications in a Multicenter Registry of Patients Discharged with Diagnosis of Acute Coronary Syndrome; CARDIAC = Anti Coagulation therapy, Age, Renal insufficiency, Drop In hemoglobin, baseline Anemia in Chinese patients; CKD = chronic kidney disease; CREDO-Kyoto = Coronary Revascularization Demonstrating Outcome Study in Kyoto; CRUSADE = Can Rapid Risk Stratification of Unstable Angina Patients Suppress Adverse Outcomes with Early Implementation of the ACC/AHA guidelines; DAPT = dual antiplatelet therapy; Ht = hematocrit; ICH = intracerebral hemorrhage; IS = ischemic stroke; MI = myocardial infarction; NSAID = nonsteroidal anti-inflammatory drugs; PAD = peripheral arterial disease; PVD = polyvascular disease; PARIS = Patterns of Non-adherence to Anti-Platelet Regimen in Stented Patients; PLT = platelet; PRECISE-DAPT = Predicting Bleeding Complications in Patients Undergoing Stent Implantation and Subsequent Dual Antiplatelet Therapy; REACH = Reduction of Atherothrombosis for Continued Health; STEMI = ST-segment elevation myocardial infarction; WBC = white blood cell count.

a Minor criterion ARC-HBR (Academic Research Consortium for High Bleeding Risk)/J-HBR.

b Major criterion in ARC-HBR/J-HBR.

In 2018, the ARC-HBR introduced the ARC-HBR criteria.^8^ The HBR criteria have been validated in the study involving 9,623 patients at tertiary care center in the United States.^17^ These criteria offer definitions for identifying patients at a high risk of bleeding by encompassing a set of 20 clinical variables, with 14 being major (conferring a BARC 3 or 5 bleeding risk of ≥4% at 1 year or associated with a risk of ICH of ≥1% at 1 year) and 6 being minor (conferring an increased bleeding risk, with a BARC 3 or 5 bleeding rate of <4% at 1 year) criteria. Patients meeting at least 1 major or 2 minor criteria were categorized as having HBR.

To verify the correlation between the criteria and bleeding outcomes, there are several attempts in validating the ARC-HBR criteria. In a post hoc analysis of the PENDULUM (Platelet Reactivity in Patients With Drug Eluting Stent and Balancing Risk of Bleeding and Ischemic Event) registry, ARC-HBR criteria were validated for 6,267 Japanese patients undergoing PCI.^18^ The analysis concluded that the ARC-HBR criteria were applicable to Japanese patients undergoing contemporary PCI and could help identify patients at risk of major bleeding. In this registry, 50.8% of patients were found to have HBR. The reported incidence of major bleeding at 12 months post-PCI were significantly elevated in patients with HBR compared with non-HBR patients, 4.2% and 1.4% (*P <* 0.001), respectively.

The different criteria and scores for evaluation of bleeding risks presented a challenge for cardiologists to select the right tool. Thus, a study aiming to compare and validate different bleeding risk scores, including ARC-HBR, PRECISE-DAPT score, PARIS score, and CREDO-Kyoto (Coronary Revascularization Demonstrating Outcome Study in Kyoto) score, for long-term bleeding outcomes in Japanese patients undergoing PCI with second-generation drug-eluting stents was conducted.^19^ It demonstrated that the ARC-HBR criteria were more sensitive in identifying patients with bleeding events compared with other risk scores. The higher sensitivity of ARC-HBR stemmed from the inclusion of additional risk factors, although this came with a trade-off between sensitivity and specificity.

Nevertheless, there remains gaps in the validity of these high bleeding risk scores in specific populations. For instance, elderly patients face a significantly elevated risk of bleeding compared with their younger counterparts.^20^ Despite the existence of various bleeding risk scores, only a few of them have been validated for use in elderly population. Likewise, validation studies have suggested that the predictive accuracy of the ARC-HBR criteria may differ between female and male patients undergoing PCI.^21^

Moreover, some of these bleeding risk scores were developed primarily in Western populations, which exhibit different ischemic and bleeding profiles compared with the East Asian population. Therefore, several ischemic and bleeding risk scores tailored to East Asian populations have been recently introduced, including the J-HBR, Asian DAPT, CREDO-Kyoto risk, and KAMIR-NIH DAPT (Korean Myocardial Infarction Registry–National Institute of Health DAPT) scores.^22^, ^23^, ^24^

The KAMIR-NIH DAPT score was developed to assist in selecting P2Y_12_ inhibitors by assessing combined ischemic and bleeding events in East Asian patients with ACS. The Asian DAPT score designed to assess the optimal DAPT duration was developed based on data of a substantial number of patients from Korean registries and was validated by trials in South Korea and Japan. The CREDO-Kyoto thrombotic and bleeding risk scores aim to distinguish ischemic and bleeding risks in the Japanese population.

Recently, the PRECISE-HBR score has been developed by integrating the PRECISE-DAPT score with the ARC-HBR criteria. This novel score offers several advantages over previous risk algorithms and has been externally validated in a large Asian cohort, supporting its applicability across different ethnicities.^25^

### High-bleeding-risk population

Various analyses were conducted in attempt to quantify the incidence of bleeding in correlation to patients with high-bleeding risks. In the TICAKOREA (Ticagrelor Versus Clopidogrel in Asian/Korean Patients with ACS Intended for Invasive Management) study involving Korean patients, the incidence of major bleeding, defined by BARC type 3 or 5 bleeding, was 10.0% and 3.7% in HBR and non-HBR patients, respectively.^26^ In a post hoc analysis for the 4-year follow-up of I LOVE IT 2 (Evaluate Safety and Effectiveness of the Tivoli DES and the Firebird DES for Treatment of Coronary Revascularization) trial that compared 6 months vs 12 months DAPT, 2,737 Chinese patients with CAD were given biodegradable or durable polymer-coated sirolimus-eluting stents and were assessed for HBR.^27^ The major bleeding incidences based on BARC type 3 or 5 levels were 2.95% and 1.52% in the HBR and non-HBR patients, respectively. HBR patients, comprising 16.0% of the trial population, had a higher risk of BARC type 3 or 5 bleeding (2.95%) and all-cause death (5.68%) at 4 years.

In a real-world cohort study aiming to validate the ARC-HBR criteria, the prevalence of patients with HBR was reported to be 44.4% among the 9,623 patients who underwent PCI.^17^ The rate of the primary bleeding endpoint at 1 year was 9.1% in HBR patients compared with 3.2% in non-HBR patients. The study also demonstrated that the presence of multiple ARC-HBR criteria had an additive prognostic value and was associated with an increased risk of both bleeding and thrombotic events, and also all-cause mortality. The incidence of bleeding from periprocedural and postdischarge periods further alluded the importance of identifying patients with HBR. The incidence of periprocedural in-hospital bleeding and postdischarge bleeding were analyzed on HBR and non-HBR patients. The incidence of periprocedural in-hospital bleeding in HBR patients was reported to be 4.8% compared with 1.4% in non-HBR patients, while the incidence of postdischarge bleeding at 1 year in HBR patients was 4.6% compared with 1.8% in non-HBR patients. This observation suggested that the risk of bleeding is sustained after discharge.

## East Asian Paradox

Aside from clinical features that affect the risks in bleeding among patients, populations of different races exhibit distinct bleeding and ischemic risks. In 2012, Jeong et al^28^ introduced the concept of “East Asian Paradox” as a unique phenomenon observed in East Asian patients with CAD. East Asians exhibit a reduced pharmacodynamic response to clopidogrel compared with Caucasians, partially and theoretically caused by the cytochrome P450 (CYP) 2C19 loss-of-function allele (carrier of at least 1 CYP2C19 loss-of-function allele: 65% in East Asians vs 30% in Caucasians).^29^ On the other hand, East Asian patients presented a lower risk of stent thrombosis following cardiovascular interventions when compared with the non-Asian population. Furthermore, East Asians have been characterized to associate with a higher bleeding risk, such as intracranial hemorrhage and gastrointestinal bleeding, and a lower incidence of ischemic risks for cardiovascular death, MI, and stent thrombosis.^30^

In a meta-analysis of 7 randomized clinical trials by Kang et al^31^ involving 16,518 patients, the clinical impact of DAPT following PCI on ischemic and bleeding outcomes was assessed, stratified by East Asian or non-East Asian patients. Notably, non-East Asians exhibited a higher incidence of major adverse cardiac events of 1.8% compared with 0.8% in East Asians, while the rate of major bleeding events were higher in East Asians (0.6% vs 0.3%). Cox proportional hazards model analysis revealed that prolonged DAPT was significantly associated with an increased risk of major bleeding in East Asians. This study suggests that the balance between ischemic and bleeding risks varies between East Asians and non-East Asians in the context of antiplatelet therapy. Prolonged DAPT may not effectively reduce ischemic events in both groups but significantly increases bleeding risk among East Asians.

Nakamura et al^32^ reviewed the high risk of bleeding in East Asian patients undergoing PCI. Japanese patients with HBR have a 3-fold higher risk of major bleeding, and approximately 50% of these patients undergoing PCI have HBR. In Japanese patients, low body weight, heart failure, and peripheral arterial disease are high-risk subsets for bleeding, and were incorporated as additional major criteria in J-HBR criteria, as a variation of the ARC-HBR criteria for Asian patients, in the Japanese Circulation Society 2020 guideline.^33^

One important limitation to acknowledge in the discussion on the varied bleeding risk in East Asian population compared with Western population is the absence of direct head-to-head comparative studies between these racial groups. Nevertheless, considering existing evidence on the potentially dire consequence of bleeding, it is essential to manage patients receiving antiplatelet therapy with regimen focusing also on the avoidance of bleeding.

### Impact of bleeding on mortality

Substantial evidence demonstrated the relationship between bleeding and mortality in patients undergoing PCI. Doyle et al^34^ reviewed the impact of major bleeding on mortality after PCI, based on data from both real-world cohorts and randomized trial populations, despite variations in bleeding definitions and patient characteristics. The impact of major bleeding on mortality after PCI was examined in several studies, and the OR ranged from 1.64 to 7.55 or HR of 1.65 to 9.96. It was suggested that bleeding may have indirect effects on subsequent adverse events that lead to increased mortality. These effects include the impact on the hemostatic response, hypercoagulability, and the discontinuation of antithrombotic medication.

The relationship between bleeding and mortality has been explored in various trials and studies. In the TRACER (Thrombin Receptor Antagonist for Clinical Event Reduction in Acute Coronary Syndrome) trial, the risk of subsequent mortality was equivalent between BARC 3b bleeding and MI and was higher following BARC 3c bleeding in patients with ACS treated with antiplatelet therapy.^35^ The APPRAISE-2 (Apixaban for Prevention of Acute Ischemic Events 2) trial, involving 7,392 high-risk ACS patients monitored for a median duration of 241 days, aimed to assess bleeding events and their subsequent impact.^36^ The study found that 2.1% of patients experienced TIMI major/minor bleeding, with those on triple therapy exhibiting a notably higher risk of bleeding compared with their counterparts on dual or single therapy. More importantly, the occurrence of TIMI major/minor bleeding events was associated with a heightened risk of all-cause mortality and ischemic events within 30 days following the bleeding event. In the outpatient setting following ACS, a systematic review of 53 studies and a participant pool exceeding 700,000 individuals also revealed similar results.^4^ The majority of bleeding occurred within 3 months after discharge and the analysis suggested a clear association between major bleeding events and increased risks of both mortality and major adverse cardiac events, underscoring the importance of monitoring bleeding complications beyond the hospital stay. Regarding in-hospital bleeding, the hemorrhagic events increased the intrahospital mortality. In a substudy of the MATRIX (Minimizing Adverse Haemorrhagic Events by Transradial Access Site and Systemic Implementation of AngioX) trial, a significant hemoglobin drop (≥3 g/dL), even in the absence of overt bleeding, has been independently associated with an increased risk of 1-year mortality.^37^ In this context, the use of bleeding risk scores in patients with ACS to predict in-hospital bleeding is recommended.^38^

The relationship between bleeding and increased risk of mortality is further explained by several potential mechanisms:

1. Altered hemostasis: Bleeding disrupts the body's ability to maintain the balance between clot formation and dissolution. This imbalance heightens the risk of thrombosis or embolism, leading to adverse cardiovascular events and elevated risk of mortality.
2. Hypercoagulability: Bleeding triggers a systemic inflammatory response, releasing proinflammatory cytokines and activating platelets and clot formation components. This heightened coagulation state substantially increases the risk of thrombotic events like MI and stroke, which can be fatal.
3. Medication discontinuation: Stopping or interrupting antithrombotic medication during bleeding episodes leaves patients vulnerable to thrombotic events. This is especially critical after coronary stenting, where these medications are essential for preventing stent thrombosis and restenosis.

In a 2-year follow-up analysis of the GLOBAL LEADERS trial, patients with bleeding event were observed to have mortality of 10.8%, and for patients experienced MI, the mortality rate was 10.4%. De-escalation of antiplatelet therapy at the time of BARC 3 bleeding was associated with a lower risk of subsequent bleeding or MI, compared with the group continuing with the same antiplatelet regimen.^20^^,^^39^

It is important to note that the relationship between the effect of the treatment on bleeding events as a surrogate endpoint and its impact on mortality, which is often the primary endpoint, has not been extensively explored in meta-analyses of clinical trials.

Gradually, the focus of post-PCI antithrombotic therapy has shifted from primarily reducing thrombotic risk to also effectively mitigating bleeding risk. The strategies for reducing or avoiding bleeding are divided into 3 key phases: before, during, and after the PCI procedure. Before the procedure, emphasis is placed on bleeding risk stratification, noninvasive testing, adherence to appropriateness criteria for revascularization, and tailoring antiplatelet therapy to avoid bleeding in high-bleeding risk patients. During the procedure, the use of radial artery access, optimal anticoagulation, and intravascular imaging-guided stent optimization are recommended. After the procedure, considerations include the duration of dual antiplatelet therapy, individualized modulation, and the use of proton-pump inhibitors to reduce gastrointestinal bleeding risk. These strategies are essential for improving the treatment outcome in patients requiring PCI.^40^ Recently, a strategy of early ticagrelor monotherapy has been investigated in clinical trials and is currently endorsed in guidelines as an effective approach to preserving ischemic protection while reducing bleeding risk after PCI in patients with ACS.^2^^,^^41^^,^^42^ The PRECISE-DAPT score has been specifically validated in patients receiving ticagrelor monotherapy, as shown in the GLOBAL LEADERS trial, where it demonstrated good predictive performance in a large, contemporary cohort.

## Nuisance Bleeding as a Prognostic Marker

Nuisance bleeding (BARC type 1), known as less severe forms of bleeding, may include symptoms such as easy bruising and bleeding of nose or gum after antiplatelet therapy.^43^ Nuisance bleeding, although minor and non–life-threatening in nature, could have significant implication for patients with CAD undergoing PCI. These minor bleedings often cause interruptions in DAPT, which is crucial for preventing thrombotic complications post-PCI. The discontinuation or interruption in DAPT caused by nuisance bleeding can increase the risk of major adverse cardiovascular events, including stent thrombosis and myocardial infarction, thereby indirectly impacting mortality. Moreover, frequent nuisance bleedings can affect patients’ adherence to prescribed antiplatelet regimens, further compounding the risk of adverse outcomes.

The risk of nuisance bleeding is high within the first year following ACS, with event rates reaching 37.5%.^4^ Nuisance bleeding is associated with certain patient characteristics and clinical factors such as older age, diabetes, and lower estimated glomerular filtration rates.^43^ These coexisting comorbidities may contribute to increased bleeding risk. Patients with nuisance bleeding within the first month of DAPT were significantly more likely to experience subsequent clinically relevant bleeding. Therefore, nuisance bleeding, as ubiquitous as it appears, should be taken as an indicator for future potential bleeding events, thus allowing clinicians to consider to preemptively de-escalate antiplatelet therapy.

## “Bi-Risk” Patients

Patients with HBR typically exhibit elevated cardiovascular risk clinical profile, a greater burden of comorbidities, and increased atherosclerotic disease extent. These characteristics often necessitate more intricate revascularization procedure or pharmacological intervention, which are linked to higher risks of future ischemic events and mortality. However, patients from real-world settings are far from homogenous, and very often, their clinical profiles include features of both high bleeding and ischemic risks. In the consensus statement on DAPT modulation by Gorog et al,^44^ a list of clinical variables and their association with bleeding risk, ischemic risk, or both were established. The clinical factors that increase both bleeding and ischemia risks include aged over 75 years, moderate/severe **c**hronic kidney disease and moderate or severe ischemic stroke in the past 6 months. These overlapping factors for both elevated bleeding and ischemic risks require extensive attention because they constitute further challenge for clinicians to manage the delicate balance between bleeding and ischemic risks.

Although there is no concrete or established definition, “bi-risk” commonly refers to specific clinical features encompassing both ischemic and bleeding risks in patients undergoing PCI. Selecting appropriate antiplatelet regimen for patients with “bi-risk” clinical features can be complicated, especially when multiple comorbidities tend to appear concurrently in frail or elderly patients. Furthermore, the choice of drug-eluting stents, procedural complexity, clinical presentation, selection of P2Y_12_ inhibitors, and concurrent anticoagulant therapy could all affect clinical decision on the treatment regimen in patients with “bi-risk” clinical features. To elucidate appropriate regimens for these patients, various analyses and clinical trials were conducted.

The recently published OPT-BIRISK (Optimal anti-Platelet Therapy for high Bleeding and Ischemic RISK patients) trial explored the use of clopidogrel monotherapy in high-risk patients with ACS who had both high bleeding and ischemic risk features and were free from adverse events for at least 6 months before randomization.^45^ Patients completed 9 to 12 months of DAPT and were then randomly assigned to either clopidogrel monotherapy or continued DAPT with aspirin and clopidogrel for an additional 9 months. Results from 7,758 patients showed that clopidogrel monotherapy led to a lower rate of BARC types 2, 3, or 5 bleeding (2.5% vs 3.3%) and a reduced rate of MACCE (the composite of all-cause death, MI, stroke, or clinically driven revascularization) (2.6% vs 3.5%) compared with dual therapy. There were no significant differences in all-cause death, MI, stroke, clinically driven revascularization, or stent thrombosis. These findings suggest that clopidogrel monotherapy may offer better bleeding outcomes without compromising ischemic risk in high-risk patients with ACS after 9 to 12 months of DAPT.

In the recent HOST-EXAM (Harmonizing Optimal Strategy for Treatment of coronary artery stenosis-Extended Antiplatelet Monotherapy) trial, 5,438 Korean patients who had been on DAPT without experiencing clinical events for 6 to 18 months following PCI with drug**-**eluting stent were randomized to receive either clopidogrel or aspirin monotherapy.^46^ The trial revealed that over a 24-month period, clopidogrel monotherapy significantly lowered the incidences of all-cause death, MI, stroke, readmission caused by ACS, and major bleeding, compared with aspirin monotherapy. In the post hoc subgroup analysis of the HOST-EXAM study, the long-term outcomes of clopidogrel monotherapy vs aspirin monotherapy were investigated in patients who had undergone PCI with drug**-**eluting stents and exhibited high thrombotic and/or high bleeding risk.^46^^,^^47^ The study demonstrated that clopidogrel maintained its benefits over aspirin in chronic maintenance for patients with CAD after PCI during 24-month follow-up, regardless of their thrombotic or bleeding risk status, emphasizing its potential as a single antiplatelet agent in these populations (absolute risk difference [ARD] 7.5% vs ARD 4.1%, with or without high thrombotic risk, respectively. ARD 9.2% vs ARD 4.0%, with or without high bleeding risk, respectively). These findings supported clopidogrel as an alternative monotherapy to aspirin in patients with varying thrombotic and bleeding risks post-PCI in chronic maintenance phase. The 2024 European Society of Cardiology guidelines for the management of CCS is recently updated to recommend clopidogrel as an alternative to aspirin monotherapy in patients with CCS with prior MI or remote PCI.^42^

## Summary

This review paper explores various critical concepts related to bleeding events in patients with CAD on antiplatelet therapy. These concepts include the prevalence of bleeding events, classifying the severity of bleeding, and a comparative review of bleeding events in East Asian and non-East Asian populations. Clinicians are increasingly adopting a "bleeding risk-first" approach, tailoring antiplatelet therapy based on factors such as age, comorbidities, and bleeding risk to optimize patient outcomes. It is essential to assess the impact of bleeding events on ischemic events and mortality in patients with CAD. Based on the cumulative studies and reports, the mechanism linking the bleeding risk, severity and ischemic events to the clinical outcome is presented (Central Illustration). Tailored risk assessment and considering region-specific factors are crucial in the management of patients with CAD in East Asian populations. Finally, selecting antithrombotic therapy in "bi-risk" patients—those with both high bleeding and ischemic risks—requires careful consideration, as evidenced by recent studies that clopidogrel monotherapy during chronic phase offers superior safety profile without compromising ischemic events compared with aspirin monotherapy or prolonged dual antiplatelet therapy in patients with ACS exhibiting both high bleeding and ischemic risks.

**Central Illustration:**
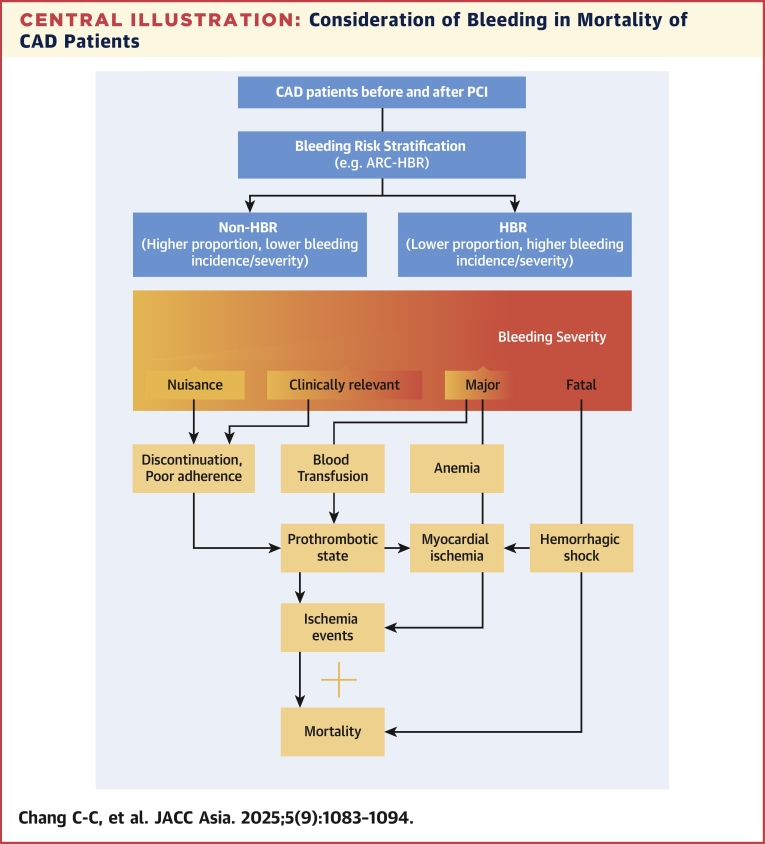
Consideration of Bleeding in Mortality of CAD Patients The presence or absence of high bleeding risk (HBR_ plays an important on the tendency and the outcome on bleeding consequences for patients with coronary artery disease (CAD) receiving antiplatelet therapy after coronary intervention. Different severity of bleeding could lead to consequences such as discontinuation or poor adherence to the prescribed antiplatelet regimen, anemia, or requirement of blood transfusion, which could lead to exacerbation of clinical status, and eventually, mortality. These events may not be mutually exclusive to each other. lead to mortality. ARC-HBR = Academic Research Consortium for High Bleeding Risk; CREDO-Kyoto = Coronary Revascularization Demonstrating Outcome Study in Kyoto; PARIS = Patterns of Non-adherence to Antiplatelet Regimen in Stented Patients; PCI = percutaneous coronary intervention; PRECISE-DAPT = Patients Undergoing Stent Implantation and Subsequent Dual Antiplatelet Therapy.

## Funding Support and Author Disclosures

This study was supported by Sanofi. The authors, individually and collectively, are responsible for all content and editorial decisions and did not receive payment from Sanofi directly or indirectly (through a third party) related to the development/presentation of this publication. This paper did not receive any specific grant from funding agencies in the public, commercial, or not-for-profit sectors. The authors have reported that they have no relationships relevant to the contents of this paper to disclose.
